# **Gender and generation perspectives in the narratives of sexually abused women in childhood** *

**DOI:** 10.1590/1518-8345.2771.3078

**Published:** 2018-11-29

**Authors:** Lucimara Fabiana Fornari, Karen Namie Sakata-So, Emiko Yoshikawa Egry, Rosa Maria Godoy Serpa da Fonseca

**Affiliations:** 1 Universidade de São Paulo, Escola de Enfermagem, São Paulo, SP, Brazil.

**Keywords:** Violence, Gender and Health, Child Abuse Sexual, Sex Offenses, Nursing, Social Media

## Abstract

**Objective:**

to analyze the narratives of sexually abused women in childhood, identifying issues related to gender and generation.

**Method:**

descriptive research with a qualitative approach, based on 214 reports selected from the Brazilian campaign #*primeiroassedio* (first harassment), which took place on Twitter social network, collected from a structured instrument. Thematic content analysis was used.

**Results:**

girls were the main victims of sexual abuse. The perpetrators were mostly male and people they knew. Five categories emerged from the narratives: Sexual abuse in the aggressors’ discourse; The child as the object of sexual pleasure; Violated childhood; Victims’ guilty feelings; and Repercussions of sexual abuse experienced in childhood.

**Conclusion:**

sexual abuse often occurs in the family context and, even if sometimes veiled, the submission of girls’ power in gender relations and of children in generation relationships is evident. Analyzing sexual abuse under the categories of gender and generation contributes to an in-depth understanding of the phenomenon, directing practices more effectively to their coping.

## Introduction

Child sexual abuse is a complex social phenomenon of great repercussion. An American study of fatal and nonfatal sexual abuse cases found that in 2015 they represented an economic cost of $9.3 billion to the US. This cost was associated with health, productivity, child welfare, violence, crime, education and quality of life^1^.

According to a report by the United Nations Children’s Fund, in 2012, an average of 17 million reports of sexually abused women in childhood were recorded in 38 low- and middle-income countries. In 28 European countries, there were approximately 2.5 million reports of adolescents sexually abused before 15 years of age^2^.

Most sexually abused children often do not report the situation due to fear, guilt, shame, lack of trust and lack of knowledge about support services^2^. However, the communication of child sexual abuse is crucial to its confrontation, since it allows for the interruption of violence, assistance to the child and their families, and the implementation of protective measures^3^.

In the year 2015, in Brazil, a social network created the campaign #*primeiroassedio* (first harassment), based on the case of a 12-year-old girl who, after participating in a television program, suffered various forms of aggression, mainly via the internet. In this campaign, women were able to report and share situations of sexual abuse experienced during childhood.

The present study used this database. Although, the campaign has the word “harassment” in the title, in this research we adopted the term “sexual abuse”. This is the way in which Brazilian legislation criminalizes the actions practiced against children and adolescents, from the carnal conjunction or libidinous act, in person or by electronic means^4^. This term is also incorporated into the Medical Subject Headings (MeSH) as the recommended terminology to approach the subject.

It is noteworthy that reports on child sexual abuse do not reveal its magnitude, since there is a significant number of sexually abused children who externalize or denounce this traumatic experience only in adult life^5^. This aspect greatly hampers further interpretations associated with the phenomenon, such as those using gender and generation cuts. In the scientific production on the subject, analyzes of child violence in these perspectives are still under construction and seek to deepen the discussions for the recognition, identification and coping of the problem^6^ ^-^ ^7^.

Aiming to contribute to the development of analyzes on child violence, as well as to provide a broader understanding of child sexual abuse, in addition to the merely descriptive approach, i.e., looking at interventions in social reality, the objective of this study was to analyze the narratives of women who had been sexually abused in childhood identifying issues related to gender and generation.

## Method

This is a descriptive research of a qualitative approach, based on reports from the users of a social network, Twitter, which establishes momentary connections. Its users use the “#” (hashtag) to highlight a subject in a tweet, which consists of a message with a maximum of 140 characters. Tweets that have the same label can be grouped and searchable on a specific topic^1^. The grouping allows the creation of campaigns through a message, usually linked to events and widely disseminated by social media.

In this research, reports were selected from the Brazilian campaign #*primeiroassedio* (first harassment), in which young women and adults published tweets about sexual abuse experienced in childhood. Data collection was performed from October 22 to November 22, 2015, one month after the creation of the hashtag.

For the collection and systematization of the data, a structured instrument was used, containing the number of the tweet, the victim sex and age, the location of the aggression, the relationship between the aggressor and the victim, the date and time of publication, and full report.

A total of 530 tweets, identified by codes from T1 (tweet 1) to T530 (tweet 530), were collected. For the research, 214 tweets were selected, considering the inclusion criteria: reports of women; age between four and nine years at the time of sexual abuse; presenting the location of the aggression; the aggressor’s sex; and relationship of the aggressor with the victim. We excluded publications that did not refer to personal reports of sexual violence and repeated messages.

The minimum age at the time of sexual abuse was established as four years due to the possibility of users remembering and describing the situation more accurately. Studies carried out with children allegedly sexually abused to verify the potentiality of the forensic interview to investigate the crimes also presented four years as the minimum age of participants^8^ ^-^ ^9^.

The reports of the selected tweets were submitted to thematic content analysis^10^. The stages of pre-analysis, exploration of the material, treatment of results, interpretation and inference were performed. The categories of analysis were Gender and Generation, anchored in the framework of historical and dialectical materialism, established by the researchers due to the potential for capturing and interpreting the researched social phenomena.

In this context, the gender category is based on the difference between men and women, which composes the social relations and the construction of meanings on the relations of power in society^11^. The generation category, in turn, is predicated on beyond the age of social subjects, insofar as it defines the social statutes of a particular group through political and ideological similarities, located in time and space^6^.

This research did not require approval from the Research Ethics Committee because it used data from a social network, of free access on the Internet. The Consolidated Criteria for Reporting Qualitative Research (COREQ) guide was applied in order to verify the scientific quality of the research. This guide was adapted according to the specificity of the research database. In this way, the criteria of the second domain were partially met and those of the third domain were completely met.

## Results

In the data collected, a total of 214 Brazilian women reported having been sexually abused in childhood. It should be noted that, due to the use of tweets, it was not possible to characterize these participants. Violations had occurred when they were between four and nine years of age. Approximately one quarter (27.57%) of the participants stated that the situation of violence had occurred at the age of eight; 21.02%, at nine years of age; 16.82%, at seven years of age; 15.88%, at six years of age; 10.74%, at five years of age; and 7.94% at four years of age. The number of cases increased as age increased.

Concerning the location of the aggressions, 48.13% of the participants reported it was at home and 13.55% in the street. The other participants mentioned the school, the church, the park, the beach, the farm, the pool, the shop, the bakery, the mall and the bus. We found that child sexual abuse is more frequent in the home environment.

Regarding the authors of the aggressions, 97.66% were male. The age of the aggressors was not reported in 84.11% of the tweets. When reported, the victims informed that the perpetrators of sexual abuse were older than the victims. As for the type of relationship with the aggressors, 22.42% were unknown persons. The other participants were victims of known persons, such as family members and friends of the family.

The analysis of the reports allowed the emergence of five empirical categories: “Sexual abuse in the aggressors’ discourse”; “The child as an object of sexual pleasure”; “Violated childhood”; “Victims’ guilty feelings”; and “Repercussions of sexual abuse experienced in childhood”.

In the category *Sexual abuse in the aggressors’ discourse,* the participants revealed that the abuse was not restricted to physical contact. The aggressors used terms that expressed desire for the developing female body: *When I was seven, a friend of my father’s, every time he saw me, he used to say I was beautiful, and that when I turned 15 I would marry him* (T204). *I remembered that when I was eight, a 16-year-old boy said that I was ‘just ready’* (T51). *At the age of eight I used to be called hottie by the masons* (T134).

The reports revealed that the aggressors expressed desire for the female body during the childhood of the victims and their adolescence was the moment for the consummation of that desire. Such a fact leads us to believe that the aggressors see the child being prepared to become an adult and to “be ready” to be “attacked.” It is as if in the phase of adolescence or of adult life the woman was allowed by society to be violated, which, in childhood, would be socially condemned.

The speeches also revealed the child’s reification (“being ready”) and a wild disposition (“attack”), in which the adult man is the predator and the strong element, while the child is the prey, the weak and helpless element of the relationship.

In the category *The child as an object of sexual pleasure*, the survey participants reported that, in the context of sexual abuse, their bodies appeared to be territories of free access to the perpetrators insofar as they were touched without consent: *When I was seven, a priest of my family was here at home. He and I were alone in the bedroom and he ran his hand over my ass. Disgusting!* (T167)*. My stepfather. He ran his hand over my body while my mother slept [...]* (T294). *A grandfather of a little friend took advantage of the fact that he was taking care of us to put his hand in the middle of my legs* (T409). *A great-uncle always kept putting me on his lap and was running his hand over me over my shorts* (T83). *In the living room of my grandmother’s house, I was alone with my uncle and he put his hand inside my panties* (T122). *A friend’s father would put me in his lap and put my hand inside my panties, touching me* (T228).

The comments emphasized the domestic environment as a frequent place for the occurrence of sexual abuse. Violations generally occurred in situations understood as caring and affection, at times when the aggressor and the child were alone.

In the category *Violated childhood*, the speeches revealed that aggressors took advantage of the naivety and innocence of children to engage in sexual abuse: *My dad made an album of me taking a shower. I was sexually suggestive in the photos, ‘in his sick mind’. I was six years old (T68). It was with a painter at my parents’ house. I was nine years old. He made me suck his member. I had no idea what I was doing* (T245). *My aunt’s husband used to caress my legs and moan as he did so. I was only four; it took me about 10 [years] to understand what it meant* (F306).

The moments of play, a characteristic activity of childhood, consisted of appropriate situations for adults to practice sexual violence in a veiled and somewhat permissive way: *At the beach house, a family friend put his hand under my dress and asked if I wanted to play (T501). The neighbor used to do a “game” throwing me and his daughter, at same age, up and letting us falling down through his body* (T386). *I was playing, and then an uncle put me on his lap and smoothed the breasts that I did not even have* (T496). *An uncle called me to play doctor. He put himself on top of me and began rubbing himself* (T168).

The aggressors took advantage of situations characteristic of childhood, such as play, to practice sexual abuse. In this way, the participants could not immediately recognize the violence, which prevented the attackers from being blamed and reported.

The category *Victims’ guilty feelings* included testimonies of participants who, when in childhood, had difficulties to report sexual abuse to relatives or persons they knew because they felt guilty. Even in situations where they tried to report what happened to some adult, they did not receive attention. In some cases, the violation was considered a reason for mockery: *My first harassment came from my stepfather when I was eight, unfortunately! I was ashamed to tell my mother, but today he is paying for everything* (T231)*. Me, nine years, swimming training. He, the driver who used to take me to the club. For years I thought it was my fault* (T471). *I was nine years old and a neighbor squeezed my breasts. I told an adult and everyone laughed. I was still blamed for what happened* (T415).

The fact that the perpetrators of sexual abuse are known adults or who have an affective bond with the participants may have further confused the perception in childhood about the abusive act.

In category *Repercussions of sexual abuse experienced in childhood,* the reports described long-term consequences for the participants’ lives. They referred mainly to psychological and social traumas, which influenced the interaction with other people and the perception about themselves: *Nine years old, a man tried to grab me in front of my cousin’s old house. To this day I do not walk the street where it occurred* (T263). *I was eight years old, a relative kissed me against my will and ran my hand all over my body. No one knows the psychological consequences of this* (T182). *Seven years old. A man grabbed hold of me inside the church. After kissing me, he apologized. I felt disgusted with myself, developed OCD (Obsessive Compulsive Disorder)* (T388). *At six, on vacation, I woke up with a friend of my brother’s hand on my ass. I was never a child anymore* (T280).

The reports show that sexual abuse in childhood consisted of a traumatic experience that had many repercussions in the life of the participants.

## Discussion

The analysis of the reports of Brazilian women who experienced sexual abuse in childhood confirms that the phenomenon is determined by unequal relations of generation associated with the constructions of gender roles, which influence patterns for the female and male since childhood^11^.

The differences between the generations and the genders, which underlie the manifestations of violence in childhood, are results of the established asymmetry of power between the female and the male (intergender relationship) and between the child and the adult (intergenerational relationship)^6^ ^-^ ^7^ ^,^ ^12^.

The fact that the participants had suffered sexual abuse between the ages of four and nine, especially in the domestic context and by men they knew, is similar to that found in other research with sexually abused children and adolescents. It was identified that the first violations were recorded in the pre-school and school phases and that the perpetrators were predominantly male and known by the victims^13^.

This aspect demonstrates the submission and dependence of the victims in relation to the perpetrators, most of whom are responsible for the victim’s subsistence. Thus, some adults responsible for the care of the child or who maintains an affective bond with her have totally distorted the love or care relationship, committing acts that implied child sexual abuse, with attitudes vastly opposed to that expected.

The domestic environment presents itself as a favorable place for perpetrating child sexual abuse, since, in general, it guarantees protection to the aggressors and the silence of the victims. Data from the Violence and Accidents Surveillance System (VIVA) Report from 2009 to 2011 reiterate the results of this study revealing that the highest proportions (62.4%) of the cases of violence against children and adolescents occurred at home^14^.

Girls suffered sexual abuse mainly from male offenders. This highlights the importance of considering the gender issues for understanding and interpreting this phenomenon. This result also corroborates the data from the VIVA Report, from 2009 to 2011, in which girls stood out among the victims of sexual violence (79.8%), and most perpetrators were male (95.5%)^14^.

The fact that girls represent the majority of victims of child sexual abuse is associated with the relationship of domination between the aggressors and the victims, forming two forms of submission: related to gender (male over female) and to generation (an adult over a child)^14^ ^-^ ^15^.

These forms of domination become more expressive when perpetrators are known to the victims, since children are often dependent on adults - usually family members - to survive. Since the family is socially recognized as responsible for providing care for the child, guaranteeing adequate conditions for child development, it is contradictory that the person who should protect and care for the children harms them. For children, acknowledging the abuse even when adults represents a double violence - the aggression itself and the refusal of protection.

On the other hand, the State, which should share with the families the responsibility for child protection, appears to be negligent both in public policies and in effective actions. Studies highlight the failures of services and the child protection network, with lack of knowledge of professionals about what to do and where to refer victims, shortage of workers and lack of support from other services. There are also gaps in professional training, lack of clarification in the definition of roles, work overload, lack of time to implement more effective care, fear of involvement with the problem and fear of invading family privacy^16^.

The reports published in the #*primeiroassedio* (first harassment) campaign also revealed that the manifestation of sexual desire by men is not limited to adult women, but extends to girls, arousing greed, situation that incurs in abuse. Therefore, the female body has become the object of sexual desire since childhood, with the foreshadowing that, at some point of its development, it can be sexually dominated.

When the victim of sexual violence is a child, there is also the difficulty to understand the abusive relationship. A survey of 118 children between five and eight years of age, using a risk scenario simulation, found that 20% of the participants accepted verbally leaving a place with an unknown person, while half of the sample reported the presence of this unknown only after having been questioned, and one-third of the children did not disclose this information after being questioned^17^.

The location and author of the aggression make it prone to perpetrate child sexual abuse, since the victim is involved in power relations associated with gender and generation issues, occupying the weakest and most powerless pole, including on the autonomy of their own body.

Concealed and hidden abuse in the private spaces of homes or in relations of friendship and kinship confuse children, who have no defense mechanisms and arguments against adult domination. This is aggravated when sexual abuse is reported to adults and disqualified.

A survey of 11,364 Finnish children about the communication of sexual abuse in childhood found that 80% of the participants had shared the situation, in which friends (48%) and mothers (20%) were the most frequent listeners. However, only 7% had reported it to the police, 5% to a teacher, 4% to a social worker, 3% to the school counselor and 2% to the school nurse. The main justification for not revealing it was to consider the experience as irrelevant (41%)^18^.

In addition, the fear, guilt and concern associated with the reaction of adults constitute barriers to reporting cases of sexual abuse experienced in childhood and adolescence. This situation can be overcome by the dialogue established for the sharing of experiences and the search for support to cope with violence^19^.

The frailty manifested by children in recognizing sexual abuse and the difficulty in distinguishing reality from fantasy were artifices used by perpetrators to commit rape during false play. Thus, the experience of being abused in childhood reflected in the play of the victims, which is one of the ways children interpret the world in which they live.

With regard to the recognition of violence, a study of 161 participants aged four to 17 years found that only from 14 years of age children were able to provide adequate terms for the sexual parts of the human body. This may be related to the lack of knowledge about the terms, the use of colloquial terms or the refusal to talk about the subject^20^. Therefore, the identification of child sexual abuse is a complex task.

The difficulty of children in understanding sexual abuse and the feelings of guilt later unleashed are generally recognized in adult life. And, of course, recognizing the situation can leave short-term and long-term marks on women’s lives^7^.

A study of 67 adult participants who had been victims of sexual abuse in childhood found three barriers to the denunciation. The first was the internal barrier, associated with guilt, shame and self-protection, as well as with immature development and the attempt to minimize effects on their own. The second barrier was related to the relationships with other people, marked by family problems, power relations with the aggressor, repercussions of the complaint and the fragility of the support network. The third referred to the social world, especially to stereotypes regarding sexuality^21^.

Another repercussion of violence against children that does not reveal the magnitude of the problem is the silence of the victims about sexual abuse, evidenced by the high number of revelations only in adult life. Thus, the longer the time elapsed for disclosure, the lower the criminalization rates of the aggressors and the greater the effects and aggravations against the health of the victims^22^.

It is well known that children who have been sexually abused experience the negative effects of this throughout life. The most common manifestations are inadequate school performance, psychological problems (depression, anxiety, suicide attempt and post-traumatic stress disorder) and personal relationships. They can also be victims in other relationships that occur in different spaces of society and present difficulties in following socially imposed norms^7^ ^,^ ^12^ ^,^ ^23^.

A study of 222 men and 660 women that had been victims of child sexual abuse found that girls were more likely (1.2-2.2 times) than boys to seek medical care because of physical health problems, such as digestive, locomotor and genitourinary symptoms. The authors describe that child sexual abuse is responsible for generating costs for the health system and highlights the need for qualified services to treat physical and mental health problems of sexually abused girls and boys^24^.

Short-term and long-term health problems triggered by the sexual abuse of girls highlight the importance of recognizing and coping with this problem through actions planned and implemented by professionals responsible for the care of children, adolescents and women in health services.

A study conducted with adults who experienced sexual abuse in childhood emphasized that care services need to recognize the barriers that prevent denunciation, since abuse usually remains hidden for a long time because the understanding about sexuality is not disclosed to children^21^.

Meeting the care needs of girls and women who had suffered sexual abuse in childhood requires the presence of a physical space in the health service that guarantees privacy and hospitality, as well as qualified professionals to establish a relationship of trust and recognition of health needs. This highlights the importance of training and improving the approach to this subject.

A Canadian study on the skills, education, and experience of professionals responsible for caring for children has found that higher education does not guarantee preparation for care practice. However, it found that participating in training courses proposed by the health service is significant when associated to the time of professional practice^25^.

In this perspective, we highlight the importance of inserting reflections and discussions on this theme in the curricula of the health, education, social assistance and justice undergraduate courses, which are somehow responsible for the assistance of sexually abused girls, adolescents and women in the childhood. In addition, it is necessary to create permanent education programs on child sexual abuse in the care and coping services in order to promote the updating and qualification of professionals regarding the laws, public policies and service protocols, which are subject to continuous changes.

Assistance to victims of child sexual abuse has been a challenge for both the professionals and the services that make up the support network, since the determination thereof lies in the historical and social construction of children and women in society. This research provides support for the understanding of how this social phenomenon is expressed among Brazilian women in order to support the development and implementation of public policies aimed at prevention and reduction of cases.

In addition, it allows health professionals, and especially nurses, responsible for serving women in different generational moments to reflect on this problem in order to incorporate, in individual or group care, questions that may give visibility to situations of sexual abuse in the childhood that remain silenced by users.

The limitations of this study are in the tweeted reports, which restrict the description of the sexual abuse experienced in childhood to 140 words and describe experiences that happened in previous years. Confirmation of the veracity of the cases consists of a limitation of the field of research that addresses testimonials of adults who had been sexually abused in childhood, since they are usually based on late memories. However, these limitations do not invalidate the study because the campaign has stimulated and facilitated the free expression of participants and the sharing of situations of child sexual abuse.

Therefore, this research presents advances in the field of knowledge about child sexual abuse by pointing issues that allow understanding the unveiling of cases in adult life, considering the perspective of gender and generation. In addition, the research provides subsidies for nurses to develop care practices that help victims to express and deal with the traumatic experience, as opposed to the re-victimization of women.

## Conclusion

This study revealed that child sexual abuse has multiple faces that could be revealed in the empirical categories that emerged from the narratives of the women who participated in the social network campaign. The results disclosed the submissive situations of gender and generation, determined by the social construction of femininity, masculinity and childhood.

The consequences of sexual violence were also highlighted, represented by physical, psychological, emotional and social damages, of acute or late character. This highlights the importance of developing and implementing measures to protect, prevent, intervene and confront this problem, which has still been veiled in society.

The creation and use of campaigns in social networks is valid for disseminating the theme and contributes to the increase of denunciations. In addition, it makes possible to understand the experiences lived by sexually abused women in childhood and can trigger responses from public policies, institutions and professionals responsible for care.

In this perspective, the Internet can be an important tool for the rapid unveiling of the facets of sexual abuse against children. However, the results of studies like this must sustain and mobilize intervention actions, qualifying institutions and professionals for the prevention and confrontation of child violence.

## Footnotes

1
^*^Available from: https://about.twitter.com/pt/company/brand-assets
